# Exposure to Atrazine during Gestation and Lactation Periods: Toxicity Effects on Dopaminergic Neurons in Offspring by Downregulation of Nurr1 and VMAT2

**DOI:** 10.3390/ijms15022811

**Published:** 2014-02-18

**Authors:** Yan Sun, Yan-Shu Li, Jun-Wei Yang, Jia Yu, Yan-Ping Wu, Bai-Xiang Li

**Affiliations:** Department of Toxicology, School of Public Health, Harbin Medical University, Harbin 150081, China; E-Mails: yansunnm@163.com (Y.S.); yanshulid@163.com (Y.-S.L.); junweiyangn@163.com (J.-W.Y.); rongrongjia1@126.com (J.Y.); ruiqingsun@yeah.net (Y.-P.W.)

**Keywords:** atrazine, orphan nuclear hormone, vesicular monoaminetransporter 2, dopaminergic neuron

## Abstract

High atrazine (2-chloro-4-ethytlamino-6-isopropylamine-1,3,5-triazine; ATR) contents in the environment threaten the health conditions of organisms. We examined the effects of ATR exposure on Sprague-Dawley rats during gestation and on the dopaminergic neurons of offspring during lactation. Pregnant dams were orally treated with 0 mg/kg/day to 50 mg/kg/day of ATR from gestational day 5 to postnatal day 22. Afterward, neither offspring nor dams received ATR. Dopamine (DA) content was examined in striatum samples by HPLC-FL; the mRNA expressions of tyrosine hydroxylase (TH), orphan nuclear hormone (Nurr1), dopamine transporter (DAT), and vesicular monoamine transporter 2 (VMAT2) in the ventral midbrain samples were examined by fluorescence PCR when the offspring reached one year of age. After the pregnant rats were exposed to ATR, the DA concentrations and mRNA levels of Nurr1 were decreased in their offspring. Decreased Nurr1 levels were also accompanied by changes in the mRNA levels of VMAT2, which controls the transport and reuptake of DA.

## 1. Introduction

Parkinson’s disease (PD) is a neurodegenerative disease characterized by the progressive degeneration of dopaminergic neurons in the substantia nigra pars compacta region of the midbrain [1]. The etiology of PD possibly involves environmental and genetic factors. However, <5% of the total cases of PD have been attributed to genetic factors [2].

Among the risk factors of PD, pesticide exposure has been associated with an increased incidence of PD among agricultural workers in rural environments [3]. Specific pesticides, such as paraquat [4], and maneb [5] have been associated with PD and another pesticide rotenone [6] can produce neurodegeneration when presented in conjunction with another toxicant. As a widely used broad-spectrum herbicide in many regions worldwide, atrazine (ATR) can persist in ground and surface water for long periods because this substance is moderately volatile and water-soluble [7].

Toxicological studies have focused primarily on the effects of ATR on the endocrine and reproductive systems [8–10]. Studies on the potential neurotoxicity of ATR have also been conducted, showing that ATR exposure can alter striatal neurochemistry; as a result, striatal dopamine (DA) levels are decreased and a loss in tyrosine hydroxylase (TH)-positive dopaminergic neurons is observed in the substantia nigra of rats [11] and mice [12]. Animal studies have also indicated that male offspring elicit anti-androgenic effects after their mothers have been exposed to ATR during pregnancy [13].

Although many PD cases are observed later in life, the pathological mechanism begins at early stages before this disease has progressed to the point at which it is diagnosed [14]. The presence of a long preclinical phase and the association of environmental exposures with an increased risk of PD have indicated that early life exposure to environmental toxicants may enhance dopaminergic neurodegeneration; for this reason, the risk of PD increases. Therefore, we considered the following questions: whether or not the toxic effect of ATR is observed on dopaminergic neurons; whether or not such toxic effects occur during the early formation and development of dopaminergic neurons; and whether or not the dopaminergic neurons of progeny are damaged if their mothers are exposed to ATR during pregnancy or lactation.

Studies on twins and relatives have shown that the susceptibility to PD may be a result of prenatal predisposition [15]. This finding suggests that genes may be involved in controlling the development and differentiation of DA neurons. Among these genes, orphan nuclear hormone (Nurr1) is considered as the most important. Studies have further indicated that Nurrl is essential for the developmental differentiation and survival of dopaminergic neurons [16]. Defects or altered expression of Nurrl in the substantia nigra (SN) is possibly associated with PD [17,18]. Hence, Nurrl is considered as a candidate gene in the etiology of PD. Therefore, we aimed to determine whether or not the appearance of PD-like symptoms is influenced by Nurr1 in rats exposed to ATR during developmental growth. We also aimed to investigate whether or not this gene functions in the neurotoxicity of ATR.

## 2. Results

### 2.1. General Status of Rats

All of the animals survived until the end of the study. No statistically significant differences were observed in body weight or food consumption at any point during the course of ATR exposure (data not shown).

### 2.2. Changes in DA Levels

**Figure 1.**
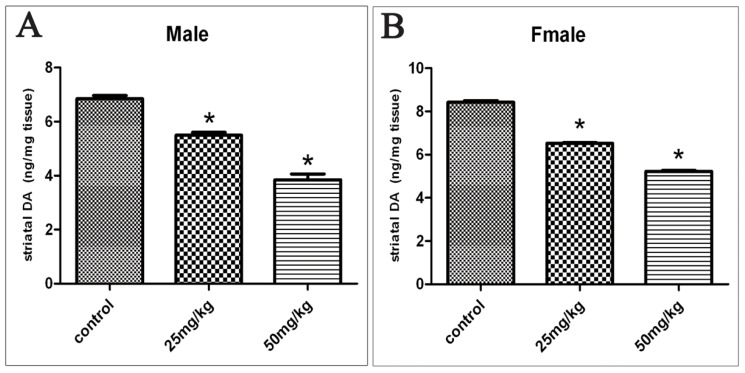
Effects of ATR on the level of DA in the striatum. Values are expressed as the mean ± SD of 10 animals per group. Mean values of DA were, respectively, 6.85, 5.49 and 3.85 ng/mg tissue in males (**A**); 8.43, 6.53, and 5.23 ng/mg tissues in females (**B**). * on top of bars indicates *p* < 0.05 (compared with the control group).

Figure 1 show the effects on striatal DA levels in the offspring exposed to ATR during gestation and lactation. In the control rats, striatal DA levels were 6.85 ± 0.37 ng/mg tissues (mean ± SD) in males. These levels decreased to 5.49 ± 0.35 and 3.85 ± 0.67 ng/mg tissues after the rats were exposed to 25 and 50 mg/kg of ATR, respectively. The striatal DA level was 8.43 ± 0.24 ng/mg tissue in the control female rats and this level decreased to 6.53 ± 0.12 and 5.23 ± 0.13 ng/mg tissue after the rats were exposed to 25 and 50 mg/kg of ATR, respectively. Striatal DA levels were decreased as the dosage of ATR was increased. Hence, a dose-dependent relationship was observed between DA levels and ATR dosage (Spearman’s Rho (*rs*) = −0.943, *p* < 0.05 in males; *rs* = −0.943, *p* < 0.05 in females).

### 2.3. Effects of Developmental ATR Exposure on Nurr1 mRNA Levels

**Figure 2.**
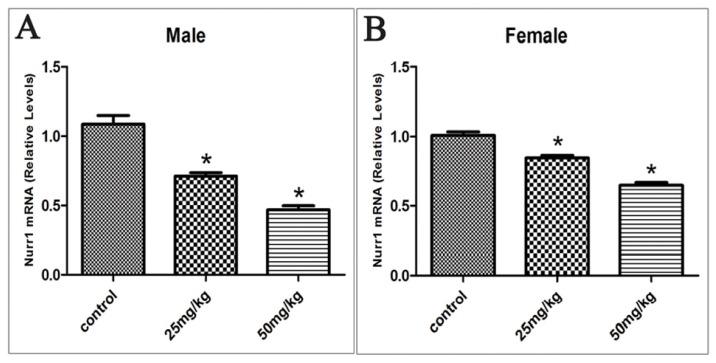
Offspring’s Nurr1 mRNA relative expression levels in the ventral midbrain in males (**A**) and females (**B**) exposed to ATR during gestation and lactation. (*n* = 10) * *p* < 0.05.

In the rat offspring exposed to ATR during gestation and lactation, Nurr1 mRNA levels decreased from 1.08 to 0.71 and 0.47 in the group of 25 and 50 mg/kg respectively in males. In females Nurr1 mRNA levels decreased from 1.01 to 0.85 and 0.65 in the group of 25 and 50 mg/kg respectively (*p* < 0.05; Figure 2). A dose-dependent relationship was also observed between the mRNA levels of Nurr1 and the dosage of ATR (*rs* = −0.934, *p* < 0.05 in males; *rs* = −0.920, *p* < 0.05 in females).

### 2.4. Effects of Developmental ATR Exposure on the mRNA Levels of TH

**Figure 3.**
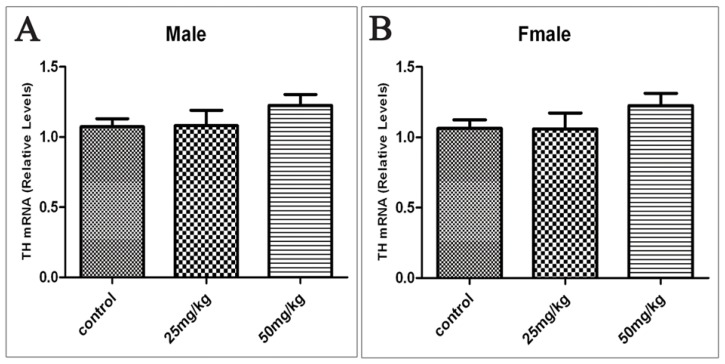
Offspring’s TH mRNA relative expression levels in the ventral midbrain in males (**A**) and females (**B**) exposed to ATR during gestation and lactation. (*n* = 10).

TH is the rate-limiting enzyme in DA synthesis. To determine whether or not the decrease in DA content in the tissues is due to ATR-induced changes in TH levels, we performed real-time PCR and analyzed the mRNA levels of TH in the ventral midbrain. The results showed no significant differences between the control rats and the ATR-treated rats (Figure 3).

### 2.5. Effects of Developmental ATR Exposure on the mRNA Levels of DAT and VMAT2

We assessed the mRNA levels of DAT and VMAT2 in male and female offspring of rats exposed to ATR during gestation and lactation after one year.

**Figure 4.**
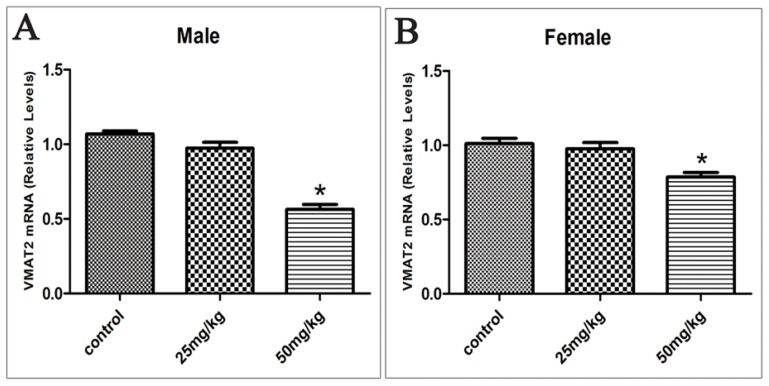
Offspring’s VMAT2 mRNA relative expression levels in the ventral midbrain in males (**A**) and females (**B**) exposed to ATR during gestation and lactation. (*n* = 10) * *p* < 0.05.

The mRNA levels of VMAT2 decreased from 1.07 to 0.97 and 0.56 in the ventral midbrain of 25 and 50 mg/kg group respectively in males. In females VMAT2 mRNA levels from 1.01 decreased to 0.98 and 0.78 in the group of 25 and 50 mg/kg respectively (*p* < 0.05; Figure 4). Hence, a dose-dependent relationship was observed between the mRNA levels of VMAT2 and the dosage of ATR (*rs* = −0.823, *p* < 0.05 in males; *rs* = −0.658, *p* < 0.05 in females).

**Figure 5.**
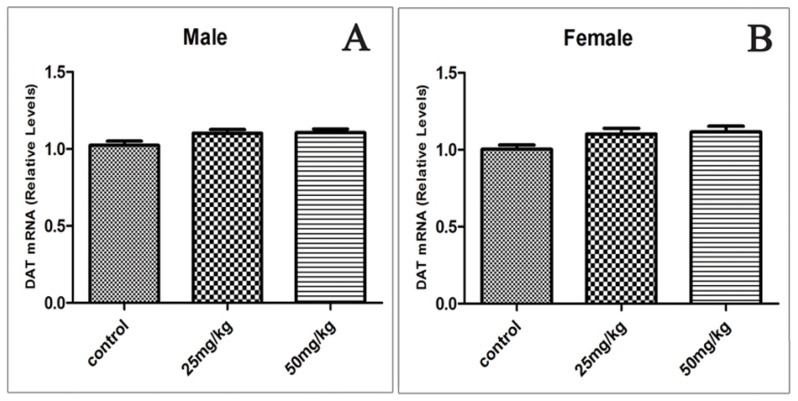
Offspring’s DAT mRNA relative expression levels in the ventral midbrain in males (**A**) and females (**B**) exposed to ATR during gestation and lactation. (*n* = 10).

An increase in the mRNA level of DAT was also observed in males (from 1.02 to 1.10 and 1.10 in 25 and 50 mg/kg group respectively) and females (from 1.00 to 1.10 and 1.11 in 25 and 50 mg/kg group respectively), but this increase was not significant (Figure 5).

## 3. Discussion

ATR is frequently detected in ground and surface water in agricultural regions because this substance exhibits limited solubility in water [19]. ATR is also one of the most frequently detected pesticides in fresh water sources in the US [20,21]. Furthermore, this herbicide has been frequently detected in rain water [22,23], fog, arctic ice, and seawater at great distances from agricultural areas [24] In living organisms, ATR is not completely removed from the body within 24 h; for instance, the major metabolites of ATR can be detected in the urine of mice at 48 h after administration [25]. Therefore, the toxic effects of ATR may be manifested only after organisms have been repeatedly exposed because this chemical has accumulated and exceeded the critical threshold.

For a short-term exposure (15 to 21 days) of a mixer-loader-tender applicator in California, the absorbed daily dose (ADD) of ATR ranges from 1.8 to 6.1 μg/kg/day [26,27]. By comparison, a higher ADD of ATR is predicted for farmers and commercial applicators in developing countries because of inappropriate personal protective equipment and unintentional excessive application. The families of ATR applicators are also at risk of high-level ATR exposure [28]. Our experimental doses administered to rats ranged from 25 to 50 mg/kg. These doses were based on the concentrations used in previous studies [13,29] and similar to 70 mg/kg/day, which is used to calculate the lowest observed adverse effect level [30].

Our study focused on the effects of ATR exposure on the development of Mesencephalic dopaminergic (MesDA) neurons. MesDA neurons control voluntary movement; as such, PD occurs when these neurons are degenerated. MesDA neurons begin to develop at embryonic day 10 (E10); at E10.5, Nurr1 expression begins shortly before TH is formed (E11.5). At E12.5, numerous MesDA neurons are formed, and development of the MesDA system continues until the postnatal period [31,32]. Ross *et al*. reported that ATR could cross the blood brain barrier and enter the brain through an unknown mechanism [25]. Rayner *et al*. [33] reported that administration of atrazine to lactating dams resulted in delayed vaginal opening in nursing litters. This study suggested that milk-derived factors might have effect on the offspring. Similar studies (Stoker *et al*., 1999; Rayner *et al*., 2007) [29,34] reported that exposure of pregnant and early postpartum dams to atrazine at 100 mg/kg/d resulted in preputial separation delays in the male offspring and resulted in increased prostate inflammation in the adult males. Stoker *et al*. also found that 14C-atrazine can be detected in the anterior and posterior hypothalamus and striatum of nursing rat when administration of 14C-atrazine a single dose of 2 or 4 mg/kg by gavage to lactating dams [35]. The above studies indicated that ATR could enter the infant brain through breast milk. In our study, the offspring were not directly treated with ATR; instead, these offspring were exposed to ATR by maternal gavage from GD5 to PND22. Neither the offspring nor the dams were treated with ATR after PND22. Hence, the duration of exposure began at the critical period of MesDA neuron development.

The present study is based on the following hypotheses: (1) exposure to ATR at early stages of life may lead to disease and dysfunction in later years [36] and (2) exposure to environmental factors during the critical periods of development can lead to adult expression of the disease [37].

DA is one of the primary neurotransmitters in the central nervous system. As such, a remarkable decrease in DA in the striatum is a sign of PD. *In vivo* [11,12] and *in vitro* [38–40] studies have shown that exposure to ATR can induce dopaminergic neurotoxicity manifested by decreased DA levels. We assessed the DA levels in the striatum of the offspring after one year of exposure of their mothers to ATR during gestation and lactation. Our studies showed that ATR can decrease the DA content in the striatum. This suggests that exposure to ATR during development can induce dopaminergic neurotoxicity.

The metabolism of DA in the brain is a complicated process involving synthesis, release, degradation, and reuptake. Dopaminergic cells in the brain use TH to convert tyrosine to levodopa (L-DOPA). L-DOPA is then converted to DA by aromatic amino acid decarboxylase (AAAD). TH is the rate-limiting enzyme for DA synthesis. Intra-neuronal DA is then transported and stored in synaptic vesicles by VMAT-2. Under normal circumstances, neuronal activation promotes the vesicular release of DA into the synaptic cleft and elicits a physiological effect via the combination of DA and DA receptors (DR). When released, DA undergoes rapid reuptake to terminate activity and maintain DA homeostasis. Reuptake is accomplished in two ways: (1) synaptosomal uptake via a DA transporter (DAT), which transports DA from the extracellular space into the cytosol; and (2) vesicular uptake by VMAT-2, which stores DA in synaptic vesicles. Free DA in the synaptic cleft is converted to homovanillic acid (HVA) by catechol-*O*-methyl transferase (COMT) and monoamine oxidase (MAO). DA in the cytosol is converted to 3,4-dihydroxyphenylacetic acid (DOPAC) by MAO [41]. If any stage of DA metabolism is disrupted, the quantity of DA is decreased, causing PD. In addition, other transcription factors, including Pitx3, Lmx1b, Nurr1, and Wnt (Figure 7), possibly affect the synthesis and metabolism of DA. Among these factors, Nurr1 was investigated in this study.

The possible reason for the decrease in striatum DA levels reported in the present studies could be an inhibition of expression of the rate-limiting enzyme for DA synthesis TH [41]. We assessed the mRNA and protein levels of TH in the ventral midbrain of the offspring after one year of exposure of their mothers to ATR during gestation and lactation. However, our studies showed that TH was not significantly affected by ATR, This is consistent with the findings of previous studies done by Filipov *et al*. [40]. Similarly, the expression of TH was not significantly affected by ATR in PC12 cells [39]. Also, TH protein levels in the striatum of mice exposed to ATR were not different among treatments at a time when DA levels were decreased [12]. This result suggested that TH was not a major target of ATR.

Another possible reason for the decrease in striatum DA levels could be the effect on transportation and storage of DA by ATR. Thus we assessed the mRNA and protein levels of DAT, and VMAT2 in the ventral midbrain of the offspring after one year of exposure of their mothers to ATR during gestation and lactation. The major finding in the present study indicated that exposure to ATR during a critical period of dopaminergic neuron development decreased VMAT2 expression in the ventral midbrain. This is similar to previous studies [14,42] that showed exposure of pregnant mice to the organochlorine pesticide heptachlor can increase the vulnerability to developing PD by influencing the expression of VMAT2 and DAT. However the difference is that the organochlorine pesticide heptachlor can upregulate the dopamine transporter (DAT) and the vesicular monoamine transporter 2 (VMAT2) in their offspring at 12 weeks of age. The possible reason is that exposure of pregnant mice to heptachlor may effect VMAT2 and DAT, and increase their compensatory expression in the adult stage, thus increasing the susceptibility to later stimulation. Results of this study showed that, with the increase of age, the aging caused the loss of expression compensatory capacity and expression downregulation, leading to DA neuron injury. The effect of ATR on the offspring in adulthood was not examined in this study.

We hypothesize that the decrease of neuronal activity in dopamine neurons by ATR may be responsible for alterations in transcription, so we determined mRNA and protein levels of the nuclear transcription factor Nurr1, known to regulate DAT and VMAT2 [43,44]. We found dose-dependent decreases in mRNA levels of Nurr1 transcription in the offspring. This indicated that, ATR could damage the DA neurons in senile rats through its early effect on Nurr1, thus changing the DAT and VMAT2 expression.

The low mRNA expression of VMAT2 reduced transport capacity and inhibited DA uptake into striatal synaptic vesicles. ATR did not exhibit a significant influence on the mRNA expression of DAT, although this expression possibly increased by 10%. Such inhibition could decrease vesicular DA levels and increase cytosolic DA levels; as a result, free DA excessively accumulates in the cytosol. Increased cytosolic DA then induces oxidative stress by producing free radicals and reactive metabolites [45,46]. Therefore, ROS-mediated toxicity occurs in DA neurons [47]. In addition, high concentrations of cytosolic DA inhibit mitochondrial respiration and can cause cytotoxicity [48].

**Figure 6.**
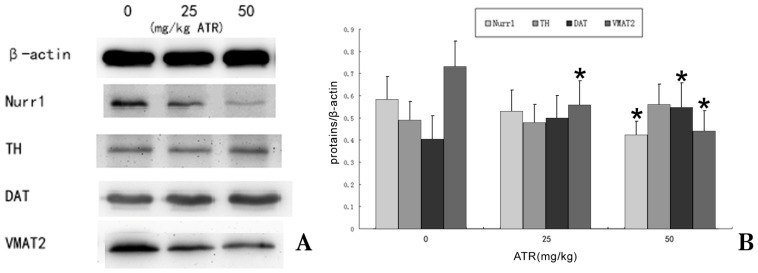
Western blot gel (**A**) and histogram (**B**) showing relative expression of Nurr1, TH, DAT and VMAT2 proteins in the midbrain. The results were expressed as the ratio of target protein/β-actin in each group. β-actin was used as the internal control. (*n* = 10) * *p* < 0.05.

To further confirm the effect of Nurr1, TH, DAT and VMAT2 on DA neurons, their protein expressions were also observed (Figure 6). Western blots showed that, the expression of Nurr1 and VMAT2 were down regulated with the increasing concentrations of ATR (0, 25 and 50 mg/kg), which had the same tendency with mRNA expression. VMAT2 mRNA decrease is only significant at 50 mg/kg while VMAT2 protein decreased significantly at 25 and 50 mg/kg. The expression of DAT was upregulated with the increasing concentrations of ATR (0, 25 and 50 mg/kg). This change tendency was also the same with mRNA, but DAT protein increased significantly at 50 mg/kg. It is possible that VMAT2 protein is lost at 25 mg/kg before a mRNA decrease is measurable. The expression of DAT protein is increased before mRNA increase is measurable.

## 4. Experimental Section

### 4.1. Chemicals and Reagents

Atrazine (CAS Registry Number: 1912-24-9; 2-chloro-4-ethylamino-6-isopropylamino-*S*-triazine, ATR, 98% purity) was obtained from Chem Services (West Chester, PA, USA). ATR solutions (25 and 50 mg/mL) were prepared by dissolving ATR in 3% starch solution.

The monoamine standards of DA were purchased from Sigma (St. Louis, MO, USA).

### 4.2. Animals and Treatment

Thirty virgin female (220 to 250 g) and thirty male (300 to 320 g) Sprague-Dawley rats were purchased from Vital River Laboratories (Beijing, China). The animals were treated in accordance with the criteria outlined in the Guide for the Care and Use of Laboratory Animals prepared by National Institutes of Health. All efforts were made to minimize the number of animals used in the experiments and their suffering.

The rats were acclimatized for one week and allowed to mate in standard stainless steel cages with one male and one female per cage. The next morning, the rats were examined for vaginal plugs. The monitoring was performed for 5 continuous days. The day on which vaginal plugs were observed was designated as day 0 of pregnancy. The pregnant rats were housed alone in standard polyethylene cages with wood shavings as bedding.

Fifteen pregnant dams were selected and randomly divided into three groups according to body weight (five rats per group). The pregnant dams received a daily dose of 10 μL/g body weight of ATR or vehicle drug by oral gavage starting on GD 5 until the offspring were weaned on postnatal day (PND) 22. The day on which the offspring were born was referred to as PND 0. Approximately 0, 25, and 50 mg/kg/day of ATR were administered (3% starch solution, vehicle control). Oral gavage was performed at approximately the same time in the morning each day.

Two female and two male weaning offspring were randomly selected from each litter. These offspring were distributed according to group and gender and then placed in separate cages until they reached one year of age.

The animals were provided purified water and food *ad libitum*. The animal cages were maintained at a constant light/dark cycle (on at 06:00 h; off at 18:00 h), temperature of 22 ± 2 °C, and relative humidity of 50% ± 15%.

After one year, the male and female offspring in the control and ART-treated groups were euthanized by administering chloral hydrate (30 mg/kg). The brains were rapidly removed and then rinsed with ice-cold saline. The midbrain and the whole corpus striatum of the bilateral side of the brain were dissected according to their different shape and location. The tissues were immediately frozen and stored at −80 °C until further processing.

### 4.3. Body Weight and Food Consumption

We recorded the body weight and food consumption of the rats once a week during the entire experiment.

### 4.4. HPLC-FL Determination of DA

The DA concentrations in the striatum were assessed using a high-pressure liquid chromatography (HPLC) system with a fluorescence detector.

The corpus striatum of the rats in each group were homogenized in 0.1 M perchloric acid and centrifuged at 10,000 rpm for 20 min at 4 °C. The homogenized samples were then filtered using a 0.2 μm cellulose membrane. The supernatants were then analyzed to determine DA contents. Afterward, the samples were injected into an Agilent chromatograph equipped with a fluorescence detector (Agilent, Santa Clara, CA, USA) and a COSMOSIL C18 Column (5 μm, 4.6 mm × 250 mm; Nacalai, Kyoto, Japan).

The mobile phase consisted of trisodium citrate (20 mM) and EDTA (0.1 mM); this pH of mobile phase was adjusted to 5.1 by adding glacial acetic acid.

Samples were separated at room temperature and a flow rate of 1.0 mL/min. The HPLC detector was set at an excitation wavelength of 285 nm and an emission wavelength of 333 nm. Data were quantified using the area under the peaks and external standards. Quantification was verified using calibration curves obtained from individual monoamine standards as reference.

### 4.5. Total RNA Extraction and Real-Time PCR

Total RNA was extracted from the ventral midbrain by using RNAiso Plus (TakaRa Biotechnology Co., Ltd., Dalian, China) according to the manufacturer’s instructions. Concentration and purity were determined by determining the absorbance at 260 and 280 nm. Approximately 1 μg of total RNA was used to synthesize cDNA (PrimeScript^®^ RT reagent kit and gDNA Eraser; TakaRa Biotechnology Co., Ltd., Dalian, China) according to the manufacturer’s protocol.

The primers of rat beta-actin, TH, DAT, VMAT2, and Nurr1 were designed using the Primer Select software program (Laser-gene, Whitehead Institute, Cambridge, MA, USA) and synthesized by TakaRa Biotechnology Co., Ltd. The primer sequences are listed in Table 1.

Target mRNA was quantified by real-time PCR (SYBR^®^ Premix Ex Taq™ II TakaRa Biotechnology Co., Ltd., Dalian, China) using an ABI 7500 Sequence Detection System (Applied Biosystems, Foster City, CA, USA). The reactions were performed in a total volume of 20 μL with 2 μL of cDNA used as a template and 10 μM of forward and reverse primers. The target sequence and beta-actin were amplified and this procedure was conducted in triplicate.

The following thermal cycling conditions were used: 30 s at 94 °C; 40 cycles of 94 °C for 5 s; 57.4 °C for 20 s and 72 °C for 20 s; and incubation at 72 °C for 1 min.

The results are expressed as relative expression ratio. The relative expression ratio of a target gene is computed, based on its real-time PCR efficiencies (*E*) and the crossing point (*CP*) difference (Δ) of one treated sample versus one control (ΔCP *control* − *treatment*). The formula is as follows:

$$\text{ratio}=\frac{{\left({\text{E}}_{\text{target}}\right)}^{\Delta {\text{CP}}_{\text{target}}\;(control-sample)}}{{\left({\text{E}}_{\text{ref}}\right)}^{\Delta {\text{CP}}_{\text{ref}}\;(control-sample)}}$$

All of the primer sets yielded a single PCR product with the expected size by agarose gel electrophoresis. Specificity was routinely monitored by checking the product melting curves (dissociation curves) in each reaction well. Standard curves were constructed using 0.5 to 100 ng of total RNA in triplicate.

### 4.6. Western Blotting

Midbrain tissues were lysed on ice for 30 min in lysis buffer containing a protease inhibitor cocktail (Roche, Baltimore, MD, USA). After centrifugation at 10,000 *g* 4 °C for 15 min, the supernatant was collected. Protein concentration was determined using a BCA protein assay kit (Applygen, Beijing, China). Equal amounts of protein (40–50 μg) were separated by 12% sodium dodecyl sulphate polyacrylamide gel electrophoresis (SDS-PAGE), electrotransferred and immobilised on a nitrocellulose membrane. The membrane was blocked with 5% non-fat milk in phosphate-buffered saline containing 0.1% Tween 20 (PBS-T) and incubated for one h at room temperature. Membranes were incubated over night at 4 °C with an appropriately diluted primary antibodies in PBS. The membranes were washed three times and incubated with horseradish peroxidase conjugated secondary antibodies for one hour at room temperature and then washed again. The targeted protein was visualized with enhanced chemiluminescent (ECL) system (GE Healthcare, Amersham Place, Little Chalfont, Buckinghamshire HP7 9NA, England, UK) according to the manufacturer’s instructions. The membranes were then exposed to CL-Xposure film (Perbio Science, Cramlington, UK). β-actin was used to normalize the samples. The densities of the specific protein bands were quantified using SIM Gel Imaging Analysis System (Bio-pro, Ashbourne, UK).

### 4.7. Statistical Analysis

We analyzed body weight and food consumption by repeated-measures ANOVA (RMANOVA; treatment × time). Dunnett’s *t* test was used to compare the body weight and food consumption of the control rats with those of the treated groups. Other data were analyzed using ANOVA. Dunnett’s *t* test was used to identify significant differences between the control group and the treatment groups. Dose-dependent relationships between groups were analyzed using Spearman correlation analysis. Data were expressed as mean ± SD. Significant effects were observed at *p* < 0.05. Statistical analyses were performed using SPSS (Chicago, IL, USA).

## 5. Conclusions

Our findings suggested a vulnerability to developing PD, in which exposure to ATR during the critical period of dopaminergic neuron development subsequently altered the dopamine system. As a result, DA levels in the striatum decreased.

A possible reason for this decrease in striatal DA levels was attributed to the decreased mRNA expression of Nurr1. This decrease may result in reduced Nurr1 activity and downregulate the mRNA expression of VMAT2. ATR would then function by interfering with the vesicular storage or reuptake of DA.

**Figure 7.**
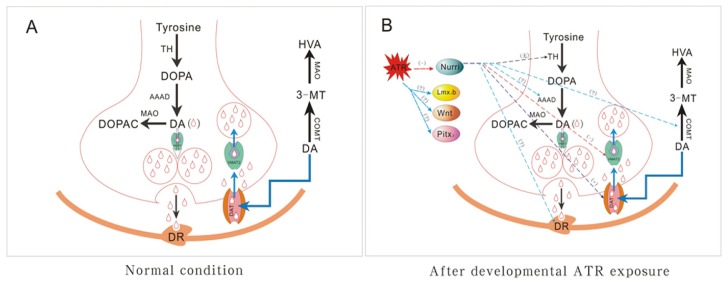
Schematic illustrating the proposed mechanism of developmental ATR exposure on dopaminergic neurons. ATR does not alter the function of TH substantially but can decrease Nurr1 transcription, which can target downstream genes, such as DAT and VMAT2, resulting in the inhibition of vesicular uptake followed by increased cytosolic DA (present study). The effects of ATR on AAAD, MAO, DR, COMT, and Pitx3 remain unknown. (**A**: Normal condition; **B**: After developmental ATR exposure.)

Thus, our findings suggested that early life exposure to ATR leads to persistent changes in the developing dopaminergic system by regulatory Nurr1 expression. This condition may increase the susceptibility to PD. Other possible ATR targets, particularly AAAD, MAO, and Pixt3, should be further investigated (Figure 7).

## Figures and Tables

**Table 1.** Primer nucleotide sequences used for real-time PCR.

Genes	Primers	Length (bp)	Annealing temperature (°C)
Nurr1	F: 5′-TGATGATCTCCATAGAGCCAGTCAG-3′ R: 5′-CCAATCCGGCAATGACCAG-3′	129	57.4
TH	F: 5′-AGCTGTGCAGCCCTACCAAGA-3′ R: 5′-GTGTGTACGGGTCAAACTTCACAGA-3′	140	57.4
DAT	F: 5′-GTACTGGCGGCTATGCTGGAA-3′ R: 5′-GGGTCTGAAGGTCACAATGCTG-3′	82	57.4
VAMT	F: 5′-CCTTCGAAGTCCACCTGCTAA-3′ R: 5′-CATCACCGATGGGATATGACTG-3′	116	57.4
β-actin	F: 5′-GGAAATCGTGCGTGACATTAAAG-3′ R: 5′-CGGCAGTGGCCATCTCTT-3′	85	57.4

## Abbreviations

ATR: Atrazine
DA: Dopamine
HPLC-FL: high-pressure liquid chromatography with a fluorescence detector
TH: tyrosine hydroxylase
Nurr1: orphan nuclear hormone
DAT: dopamine transporter
VMAT2: vesicular monoaminetransporter 2
PD: Parkinson’s disease
SN: substantia nigra
GD: pregnancy day
PND: postnatal day
Ct: cycle number
ADD: absorbed daily dose
L-DOPA: levodopa
AAAD: aromatic amino acid decarboxylase
DR: dopamine receptors
DAT: dopamine transporter
HVA: homovanillic acid
COMT: catechol-O-methyl transferase
MAO: monoamine oxidase
DOPAC: 3, 4-dihydroxyphenylacetic acid
ROS: reactive oxygen species
